# Metabolic dysfunction-associated steatotic liver disease: ferroptosis related mechanisms and potential drugs

**DOI:** 10.3389/fphar.2023.1286449

**Published:** 2023-11-09

**Authors:** Baoqiang Zhu, Yuankui Wei, Mingming Zhang, Shiyu Yang, Rongsheng Tong, Wenyuan Li, Enwu Long

**Affiliations:** ^1^ Department of Pharmacy, Sichuan Academy of Medical Sciences & Sichuan Provincial People’s Hospital, School of Medicine, University of Electronic Science and Technology of China, Chengdu, China; ^2^ School of Pharmacy, Southwest Medical University, Luzhou, Sichuan, China

**Keywords:** metabolic disease, ferroptosis, therapeutic drugs, metabolic dysfunction-associated steatotic liver disease (MASLD), mechanism

## Abstract

Metabolic dysfunction-associated steatotic liver disease (MASLD) is considered a “multisystem” disease that simultaneously suffers from metabolic diseases and hepatic steatosis. Some may develop into liver fibrosis, cirrhosis, and even hepatocellular carcinoma. Given the close connection between metabolic diseases and fatty liver, it is urgent to identify drugs that can control metabolic diseases and fatty liver as a whole and delay disease progression. Ferroptosis, characterized by iron overload and lipid peroxidation resulting from abnormal iron metabolism, is a programmed cell death mechanism. It is an important pathogenic mechanism in metabolic diseases or fatty liver, and may become a key direction for improving MASLD. In this article, we have summarized the physiological and pathological mechanisms of iron metabolism and ferroptosis, as well as the connections established between metabolic diseases and fatty liver through ferroptosis. We have also summarized MASLD therapeutic drugs and potential active substances targeting ferroptosis, in order to provide readers with new insights. At the same time, in future clinical trials involving subjects with MASLD (especially with the intervention of the therapeutic drugs), the detection of serum iron metabolism levels and ferroptosis markers in patients should be increased to further explore the efficacy of potential drugs on ferroptosis.

## 1 Introduction

Metabolic dysfunction-associated fatty liver disease (MASLD), also referred to as non-alcoholic fatty liver disease (NAFLD) or metabolic dysfunction-associated fatty liver disease (MAFLD), encompasses both simple hepatic steatosis and metabolic dysfunction-associated steatohepatitis (MASH), previously known as non-alcoholic steatohepatitis (NASH). Some cases of MASLD can progress to liver fibrosis, cirrhosis, and even hepatocellular carcinoma. In 2020, an international expert group recommended renaming NAFLD to MAFLD based on previous research and clinical evidence (Eslam et al., 2020). However, in June 2023, NAFLD was renamed MASLD again based on Multi-society Delphi consensus (Rinella et al., 2023). Currently, MASLD is recognized as a “multisystem” disease that coexists with metabolic disorders and hepatic steatosis. Metabolic diseases are characterized by at least one of the following: obesity/overweight, type 2 diabetes mellitus (T2DM), hypertension or dyslipidemia. The diagnosis of fatty liver is based on liver biopsy histology and imaging examinations indicating the presence of hepatic steatosis. The two renames of NAFLD have gradually expanded the diagnostic criteria for metabolic diseases (from including at least two metabolic diseases to including at least one of them), emphasizing the strong connection between fatty liver and metabolic diseases, and highlighting the significance of controlling the progression of metabolic disorders in the treatment and management of MASLD. Currently, a combination of drugs, such as hypoglycemic drugs, and antioxidants, has been recommended for comprehensive control and management in clinical practice (Yin et al., 2023).

Ferroptosis is a newly discovered form of programmed cell death resulting from abnormal intracellular iron metabolism, which leads to iron overload and lipid peroxidation. The main factors contributing to the development of ferroptosis include abnormal iron metabolism or excessive iron intake, oxidation of unsaturated fatty acids, and impairment of antioxidant repair mechanisms (Li J. et al., 2020). Recently, ferroptosis has gained considerable attention in medical research due to its involvement in various conditions such as cancer, diabetes, cardio-cerebrovascular disease, and liver disease (Cheng et al., 2023; Miao et al., 2023; Zhang et al., 2023). Importantly, existing evidence suggests an interrelation between metabolic diseases, fatty liver, and ferroptosis, with the latter playing a crucial role in their pathogenesis. Therefore, targeting ferroptosis may serve as a common therapeutic approach for both metabolic diseases and fatty liver, potentially improving the progression of MASLD. Based on these considerations, we have conducted a comprehensive review of current research on fatty liver, metabolic diseases, and ferroptosis, and explored the potential of relevant drugs as therapeutic interventions, aiming to provide new insights and references for readers in this field.

## 2 Ferroptosis in MASLD

**FIGURE 1:**
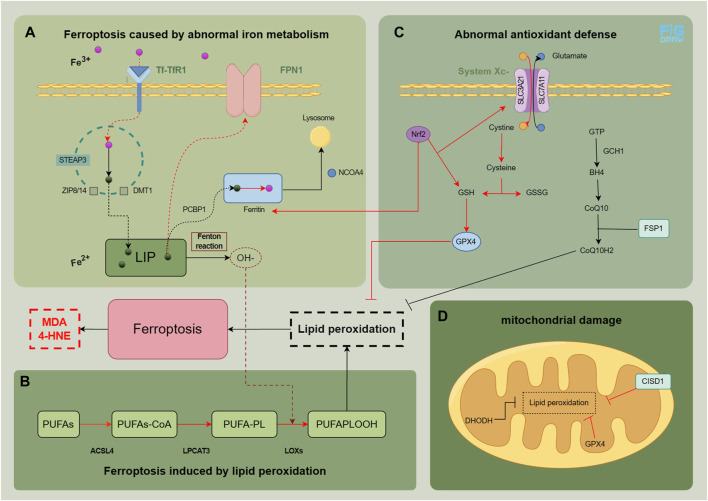
Physiological and pathological mechanisms related to ferroptosis in MASLD. In normal cells, the intake and metabolism of iron, esterification of unsaturated fatty acids, and clearance of peroxides are maintained in a relatively stable state. The main factors contributing to the development of ferroptosis are abnormal iron metabolism **(A)**, lipid peroxidation **(B)**, as well as abnormal antioxidant defense **(C and D)**. The red pathway indicates an abnormality in the process. The figure was drawn by Figdraw.

Ferroptosis, is a novel cellular mechanism of damage, characterized by iron overload and lipid peroxidation resulting from aberrant intracellular iron metabolism. The physiological and pathological processes involved primarily encompass the following four aspects. We have summarized the physiological and pathological mechanisms related to ferroptosis in MASLD (Figure 1) and summarized relevant research evidence.

### 2.1 Ferroptosis caused by abnormal iron metabolism

Iron metabolism is a complex process. Under normal conditions, transferrin (Tf) in plasma binds to transferrin receptor 1 (TfR1), facilitating the entry of Fe3^+^ into the cell (Yu et al., 2020). The six-transmembrane epithelial antigen of the prostate 3 (STEAP3), a member of the metal reductase family, acts as a ferrous reductase activity and converts Fe^3+^ to Fe^2+^. This Fe^2+^ is transported to the cytoplasm through divalent metal transporter 1 (DMT1) and zinc transporter 8/14 (ZIP8/14), resulting in the formation of a labile iron pool (LIP) (Frazer and Anderson, 2014). Free Fe^2+^ can be transferred to ferritin, with ferritin heavy chain 1 (FTH1) playing a pivotal role, through poly (rC) binding protein 1 (PCBP1), and it can be converted back to Fe^3+^ to maintain intracellular iron balance or be transported to the mitochondria for utilization (Protchenko et al., 2021). Ferroportin 1 (FPN1) enables the export of Fe^2+^ to the extracellular space. Nuclear receptor coactivator 4 (NCOA4) regulates ferritin degradation in lysosomes during this process (Mancias et al., 2014). Disruptions or imbalances in certain metabolic pathways can trigger ferroptosis when iron metabolism is disrupted. Increased ferritin degradation, elevated TfR1 expression, or decreased FPN1 expression can lead to intracellular Fe^2+^ accumulation, which generates hydroxyl peroxides through the Fenton reaction with peroxides (Haschka et al., 2021). Polyunsaturated fatty acid (PUFA)-phospholipids (PL) become susceptible targets, resulting in the production of a substantial amount of peroxides.

Multiple clinical studies have uncovered anomalies in intestinal iron absorption and iron metabolism levels among patients afflicted with MASLD. Hoki et al. conducted an oral iron absorption test, which demonstrated an augmented uptake of intestinal iron absorption in individuals with NASH. This increase is attributed to the upregulation of DMT1 expression, regulated by iron regulatory proteins (Hoki et al., 2015). The concentration of serum serves as a crucial indicator of iron reserve and *in vivo* iron metabolism. Real-world studies have consistently revealed a positive correlation between serum ferritin levels and the incidence rate of MASLD (OR:1.725, 95%CI:1.427–2.085, *p* < 0.001) (Wang J-W. et al., 2022).

Furthermore, the occurrence of MASLD is significantly more prevalent in individuals with hyperproteinemia and those adhering to a high iron intake diet in comparison with normal individuals (*p* < 0.001) (Yang et al., 2019). Patients with hyperproteinemia exhibit more severe disruptions in lipid and glucose metabolism, alongside higher levels of transaminase levels (*p* < 0.05). Histopathological analysis reveals a positive correlation between increased serum ferritin levels and the severity of steatosis and iron staining (*p* < 0.05) (Wang Q. et al., 2022).

Moreover, a double-sample Mendelian randomization study utilizing an open genome-wide association research database substantiates the association between heightened genetic prediction of liver iron and an elevated risk of MAFLD (odds ratio: 1.193, 95% CI: 1.074–1.326, *p* = 0.001). The study also establishes a significant connection between hereditarily predicted elevated serum ferritin levels predicted by heredity and MAFLD (Dataset 1: *β* = 0.038, 95% CI: 0.014–0.062, *p* = 0.002; Dataset 2: *β* = 0.081, 95% CI: 0.025–0.136, *p* = 0.004) (He et al., 2022).

Previous preclinical studies have also investigated the potential relationship between iron intake and MASLD. In one study, the progression of liver fat lesions in HFE mice subjected to a high-calorie diet with iron deficiency was compared to those on a normal iron high-calorie diet. The findings revealed that mice with iron deficiency exhibited lower liver weight and diminished expression of iron transporters and iron regulatory genes (Crawford et al., 2021). Additionally, levels of 4-hydroxynonenal (4-HNE) and malondialdehyde (MDA), which are markers of ferroptosis, were significantly elevated in hepatocytes induced by a high iron diet.

Furthermore, another preclinical study utilizing a hepatocyte-specific Trf knockout mice (Trf LKO) model discovered that feeding mice a high iron diet increased the incidence of ferroptosis-induced liver fibrosis (Yu et al., 2020). However, the administration of a ferroptosis inhibitor, ferrostatin-1 (Fer-1), effectively reversed liver fibrosis in this model. In addition, relevant studies have shown that iron overload related ferroptosis is associated with upregulation of TfR1, DMT1 expression, and downregulation of FPN1 expression (Wang et al., 2021a; Liu et al., 2022).

### 2.2 Ferroptosis induced by lipid peroxidation

The excessive synthesis of PUFA-PL can serve as a substrate for hydroxide ion (OH^−^) to facilitate the generation of lipid peroxides, inducing ferroptosis (Gan, 2022). This process primarily involves PUFAs, PL, and crucial enzymes such as long-chain acyl CoA synthase family member 4 (ACSL4), lysophosphatidylcholine acyltransferase 3 (LPCAT3), lipoxygenase (LOXs), etc (Yuan et al., 2016a; Lee et al., 2021).

Previous studies have shown that elevated arachidonic acid metabolism promotes the occurrence of liver ferroptosis in mice (Li X. et al., 2020; Tong et al., 2023), and upregulation of LOX15 (25) was observed in MAFLD mice fed on a high-fat diet. Additionally, gut microbiota metabolites also promoted the expression of ACSL4 and induced ferroptosis. The iron chelating agent effectively controlled this effect (Liu et al., 2022).

### 2.3 Abnormal antioxidant defense

The cell possesses antioxidant defense systems that regulate lipid reactive oxygen species (ROS) and lipid peroxides to counteract ferroptosis. These defense mechanisms include.

1. Glutathione peroxidase 4 (GPX4): an endogenous enzyme that removes lipid peroxides. GPX4 is synthesized using glutathione as its precursor. The Xc^−^ System transports cystine into the cell, which undergoes a series of reactions to generate glutathione (GSH), further synthesizing GPX4. The Xc^−^ System-GSH-GPX4 axis is typically inactive during ferroptosis (Chen et al., 2021).
2. Kelch-like epichlorohydrin-associated protein-1 (Keap1)-nuclear factor erythroid 2-related factor 2 (Nrf2) pathway: this intracellular antioxidant pathway regulates the expression of downstream antioxidant factors, including heme oxygenase 1 (HO-1), to mitigate ferroptosis (Song and Long, 2020).
3. Guanosine triphosphate cyclization hydrolase 1 (GCH1)/tetrahydrobiopterin (BH4)/dihydrofolate reductase (DHFR) pathway: this pathway promotes coenzyme Q (CoQ) synthesis, inhibits lipid peroxide accumulation, and provides resistance against ferroptosis (Kraft et al., 2020).
4. Ferroptosis suppressor protein 1 (FSP1): FSP1, as an oxidoreductase, reduces CoQ to CoQH2, an antioxidant that scavenges lipophilic free radicals, thus preventing lipid peroxide accumulation (Bersuker et al., 2019).
5. Mitochondrial defense system: mitochondria have defense mechanisms to against ferroptosis. For example, mitochondrial ferritin reduces iron content by storing iron, GPX4 and dihydroorotate dehydrogenase (DHODH) in the mitochondria eliminate lipid peroxides (Mao et al., 2021), and CDGSH iron sulfur domain 1 (CISD1) and voltage-dependent anion channel (VDAC) regulate iron concentration and respiratory substrate content in mitochondria, respectively (Lemasters, 2017; Lipper et al., 2019).

Two studies evaluated the progression of NAFLD using mice fed a high-fat diet (Qi et al., 2020; Ding et al., 2023) and a methionine-deficient diet (Li X. et al., 2020), indicating that elevated arachidonic acid metabolism promoted the occurrence of liver ferroptosis in mice, with the accumulation of lipid peroxides, an increase in mitochondrial reactive oxygen species, and changes in mitochondrial morphology. Meanwhile, in animal models of fatty liver, ferroptosis leads to a decrease in liver GPX4, an increase in 12/15-LOX (34), and a decrease in Nrf2(35).

### 2.4 Ferroptosis induced by other pathways

Besides these primary physiological and pathological mechanisms, recent studies have identified key factors involved in iron metabolism significantly impact ferroptosis. For instance, the p53 protein has been found to inhibit ferroptosis by suppressing dipeptidyl peptidase 4 (DPP4) and promote ferroptosis by inhibiting the solute carrier family 7—member 11 (SLC7A11) gene. This dual regulatory function of p53 holds great potential for cancer treatment (Kang et al., 2019). Additionally, ROS accumulation induces endoplasmic reticulum stress, affecting the activity of peroxisome proliferator-activated receptor (PPAR) (Yakubov et al., 2023) and influencing iron metabolism through inflammatory signaling pathways (Cheng et al., 2021a; Zhao et al., 2023). Moreover, as research progresses, emerging potential targets and receptors are being identified. The farnesoid X receptor (FXR) (Kim et al., 2022) and AMP-activated protein kinase (AMPK) (Lee et al., 2020; Wang et al., 2022c) have emerged as potential key targets of ferroptosis. For example, it has been suggested that a high-fat diet can impact Nrf2 levels by inhibiting AMPK-mediated mechanistic target of rapamycin (mTOR) activation (Liu et al., 2023). These findings enhance our understanding of the complex mechanisms underlying ferroptosis and provide potential avenues for therapeutic intervention.

## 3 Ferroptosis in metabolic diseases

### 3.1 T2DM

T2DM is primarily characterized by insulin resistance. A meta-analysis indicates that fatty liver has a prevalence of 55.5% (95% CI, 47.3–63.7) among T2DM patients (Younossi et al., 2019a). In the liver of T2DM patients, insulin resistance can lead to fat accumulation and accelerate the progression of MASLD. Recent studies have demonstrated the impact of diabetes on the liver by examining ferroptosis. For example, a study observed the pathological changes in the livers of male C57BL/6 mice with diabetes. The findings revealed fibrotic symptoms, increased levels of inflammatory and oxidative stress markers, and weakened activity of the antioxidant system in the livers of diabetic mice. Treatment with Fer-1 effectively reversed and delayed the adverse liver outcomes in diabetic mice, as evidenced by improvements in alanine transaminase (ALT) and triglyceride levels, enhanced liver antioxidant systems such as Nrf2 and GPX4, and reduced levels of interleukin-6 (IL-6) and tumor necrosis factor *a* (TNF-α) (Younossi et al., 2019a).

In addition to insulin resistance, pancreatic *β* cell dysfunction and injury are also pathological features of T2DM. Excessive iron deposition in the cells can contribute to pancreatic dysfunction (Coffey and Knutson, 2017). Iron metabolism in the body is associated with the development of T2DM. A meta-analysis summarizing the results of 12 case-control and cohort studies found a significant correlation between elevated serum ferritin levels and the prevalence of T2DM (OR = 1.43, 95% CI: 1.29–1.59) (Liu et al., 2020). Furthermore, conditions like thalassemia and hemochromatosis, which can result in iron overload, are linked to pancreatic *β* cell damage and insulin resistance (Noetzli et al., 2012; Pelusi et al., 2016). Iron, an essential component of ferrum-sulfur (Fe-S) clusters, plays a crucial role in the mitochondrial oxidative synthesis, processing and secretion of insulin (Marku et al., 2021). Disruption of intracellular iron metabolism can interfere with these metabolic processes and induce ferroptosis, leading to a decrease in insulin secretion (Bruni et al., 2018). A study (Wei et al., 2020) suggests that arsenic can induce ferroptosis by mediating ferritin autophagy in pancreatic *β* cells . NCOA4, as a selective receptor for ferritin autophagy, mediates intracellular iron transport to autophagosomes by binding to FTH1, ultimately releasing Fe^2+^, and this damage may depend on an increase in mitochondrial reactive oxygen species (MtROS). The comparative experiment of using streptozotocin (STZ) and Fer-1, the inducer of diabetes, in male C57BL/6 mice, and the comparative experiment of using erastin and Fer-1 in human islet cell clusters also have proved the relationship between ferroptosis and pancreatic *β* cells (Li and Leung, 2020). Meanwhile, research has found that under high glucose conditions, the expression of GPX4 in pancreatic *β* cells is inhibited, the synthesis of GSH is reduced, leading to ferroptosis (Li D. et al., 2020; Krümmel et al., 2021). Xc^−^ system is also necessary for insulin synthesis and secretion (de Baat et al., 2023). Based on the above research, Xc^−^ System-GSH-GPX4 axis may be crucial for clearing lipid peroxidation and maintaining normal homeostasis in pancreatic β cells. Additionally, high glucose, hydrogen peroxide, and STZ induced increased intracellular ROS, decreased activity of the Nrf2-GPX4 pathway, and reduced mitochondrial membrane potential in Rin-5F cells (Frey et al., 2020; Stancic et al., 2022).

### 3.2 Overweight/obesity

Obesity, a metabolic disorder characterized by excessive fat accumulation and storage in the body, is closely associated with an increased prevalence of metabolic diseases. MASLD represents a comprehensive manifestation of obesity and metabolic syndrome in the liver. A study in France revealed that over 20% of obese individuals with metabolic syndrome suffered from NASH(55). The expression of inflammatory cytokines in the liver and white adipose tissue may play a crucial role in the molecular signaling pathway that links obesity to fatty liver. Chronic low-level inflammation associated with obesity contributes to the production of inflammatory cytokines including IL-6 and TNF-α(56), which may directly result in liver pathology *via* endocrine mechanisms. Various adipokines produced by adipose tissue, including leptin, adiponectin, and others (Britton et al., 2016; Jorge et al., 2018), have been shown to influence the development of MASLD. Iron homeostasis is significantly connected to adipose factors, and high iron is an important negative regulator of both leptin and adiponectin (Fernández-Real et al., 2015; Harrison et al., 2023). A study using a mouse model of iron overload induced by an iron-rich diet revealed an upregulation of adipose factor levels associated with insulin resistance (Dongiovanni et al., 2013). Meanwhile, the reduction of FPN in adipocytes can lead to iron load, decreased adiponectin, and insulin resistance, which can further affect the metabolism of other organs (Gabrielsen et al., 2012). It was accompanied by a notable reduction in adipocytes as well as a potential correlation with the proliferation and hypertrophy of visceral adipose tissues. Regarding mitochondrial function, obesity can lead to mitochondrial dysfunction, which mainly occurs in the liver, muscles, and adipose tissue. The morphology and quantity of mitochondria have also changed: mitochondria in skeletal muscles have become smaller and shorter, mitochondria in white adipose tissue are small and slender, and cristae are irregular (Zhang S. et al., 2022). GPX4, a crucial enzyme for the maintenance of lipid peroxidation levels, plays a vital role in the inhibition of ferroptosis. A recent study (Schwärzler et al., 2022) found that mice with specific GPX4 deficiency in adipose tissue exhibited an increase in the number of white adipocytes and the level of serum TNF-α. In isolated adipocytes with impaired GPX4 activity, there was an increase in the level of 4-HNE and the production of inflammatory factors such as TNF-α, IL-1β, and IL-6. Furthermore, obese mice fed a high-fat diet showed a significant decrease in GPX4 expression (Schwärzler et al., 2022). In addition, obese mice with GPX4 deficiency also displayed lipid peroxidation in the liver (Katunga et al., 2015). Based on these findings, it could be inferred that GPX4 may play a role in the inhibition of lipid peroxidation, the prevention of adipose tissue inflammation, as well as the mitigation of low-level systemic inflammation. In addition, ACSL4 may have effects such as promoting the participation of arachidonic acid in phospholipids, causing liver fat accumulation, and triggering inflammation of white adipose tissue (Chen et al., 2023). For example, specific knockout of ACSL4 in mouse adipocytes effectively prevented obesity induced by a high-fat diet and reduced levels of lipid peroxidation product 4-HNE (Killion et al., 2018).

### 3.3 Other metabolic disorders

Recently, hypertension and dyslipidemia are also considered as the common metabolic disorder of MASLD. Although there is less connection between the two diseases and liver steatosis, ferroptosis plays a role in some pathogenesis of hypertension and atherosclerosis induced by hyperlipidemia. Targeted therapy for ferroptosis may be a promising new therapy.

Dyslipidemia is closely associated with hepatic steatosis and can serve as an independent predictor (Zou et al., 2021). Daily consumption of a high-fat diet exacerbates the metabolic burden on the liver, leading to the accumulation of hepatic lipids. Lipid-lowering therapy and a diet aimed at reducing fat intake are recommended management measures for MASLD. Conversely, MASLD can contribute to the development of dyslipidemia and is correlated with vascular calcification, significantly increasing the risk of cardiovascular disease. Dyslipidemia impacts the function of vascular endothelial cells and vascular smooth muscle cells, thereby promoting the development of atherosclerosis. Furthermore, iron overload and ferroptosis have been observed in vascular endothelial cells within atherosclerotic lesions.

Hypertension is now recognized as a risk factor for MASLD. Additionally, hypertension represents one of the primary clinical outcomes of MASLD, demonstrating a strong correlation between these two conditions. Recent studies have revealed that angiotensin II, a key factor in the development of hypertension, can induce astrocytes to secrete inflammatory cytokines, promote ferroptosis, and elevate the levels of ferroptosis markers. The involvement of angiotensin II in various pathological processes, including cardiac remodeling, myocardial hypertrophy, and ischemic reperfusion injury, has been observed in hypertensive mice.

**FIGURE 2:**
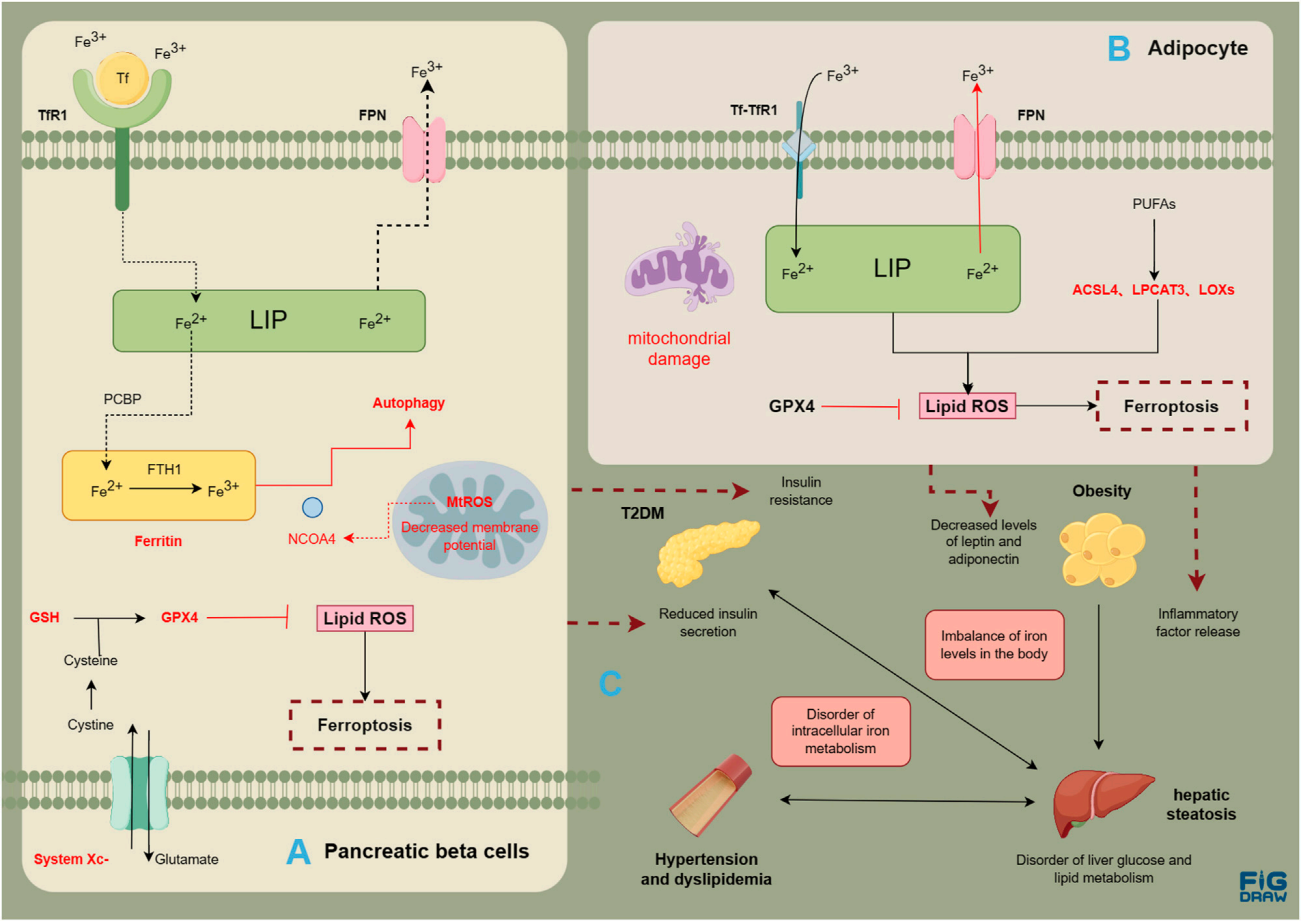
Risk association between MASLD and metabolic diseases. Evidence of ferroptosis in pancreatic β cells **(A)**; Evidence of ferroptosis in adipocytes **(B)**; The correlation between metabolic diseases and liver steatosis **(C)**. The figure was drawn by Figdraw.

MASLD is a complex condition involving multiple systems. Obesity, T2DM, hyperlipidemia, and fatty liver disease can interact with each other, thereby contributing to the development of comorbidities and collectively increasing the risk of cardiovascular disease (Figure 2). Ferroptosis is related with the normal functioning of pancreatic *β* cells, hepatocytes, vascular endothelial cells, as well as adipocytes. The disruption in various systems further results in the imbalance of glucose and lipid metabolism, with multiple organs involved, promoting the onset and progression of MASLD. It seems that ferroptosis is intricately intertwined with the pathogenic processes of metabolic diseases and MASLD. Metabolic diseases are often accompanied by disturbances in iron metabolism, a condition termed dysmetabolic iron overload syndrome (DIOS). DIOS primarily occurs in overweight individuals, such as those with type 2 diabetes, potentially progressing to MASLD.

## 4 Research on targeted ferroptosis related to clinical therapeutic drugs

Relevant studies primarily focus on the inhibition of ferroptosis in MASLD. There are three main approaches. Firstly, it involves reducing intracellular iron overload by either decreasing excessive iron intake or regulating iron metabolism. Secondly, it entails enhancing the defense system against lipid peroxidation and increasing the expression of antioxidant factors. Lastly, it involves the regulation of the activity of key enzymes implicated in the lipid oxidation process, accompanied by the modulation of the unsaturated fatty acid metabolism. Key proteins targeted in these approaches include ACSL4, GPX4, and Nrf2, among others. In this article, we provided a summary of the mechanisms of anti-ferroptosis drugs that have demonstrated efficacy in clinical trials for MASLD and other liver diseases. Our goal was to analyze and explore the commonalities and potential therapeutic targets among these drugs in MASLD treatment using different targets (Table 1).

**TABLE 1:** The mechanism of targeting ferroptosis with therapeutic drugs in clinical research.

Drug	Animal/Cell	Effect in hepatocyte	Effect in other cells
Hypoglycemic drugs			
Rosiglitazone (Wei et al., 2020)	Male Sprague-Dawley rats, L-02 cells	ACSL4↓	
Pioglitazone (Yuan et al., 2016b; Duan et al., 2022; Liang et al., 2022; Qi et al., 2023)	Male SPF/ICR mice (Liang et al., 2022), Male SD rats (Duan et al., 2022), neuronal cells (Duan et al., 2022; Liang et al., 2022)		Neurons: PPAR-γ ↑ (collaborate with Nrf2); COX2↓(73, 74)
	The human HCC cell lines HepG2 and Hep3B (75)	Stable Fe-S clusters in CISD1	
	Male C57BL/6N mice (Qi et al., 2023)		kidney tissue: GPX4↑; Stable Fe-S clusters in CISD1
Liraglutide (An et al., 2022; Song et al., 2022a)	Db/db mice and non-diabetic littermate db/m Mice, Human hepatoma HepG2 cells (Song et al., 2022a)	TfR1, NOX4↓; FPN1, SLC7A11, Nrf2/HO-1, GPX4, GSH ↑	
	Male diabetic db/db mice and nondiabetic littermate db/m Mice (An et al., 2022)		Neurons: mitochondrial transferrin, FPN1, FTH1, SLC7A11, GPX4↑; ACSL4, TfR1, mitochondrial ferritin↓
Vildagliptin (Zhang et al., 2022b)	Male C57BL/6 mice		Neurons: GPX4↑
Vildagliptin, alogliptin (Xie et al., 2017)	Human CRC cell lines (HCT116, SW48, CACO2, DLD1, and SW837), mice		Colorectal cancer cell: DPP4↓
Metformin (Ma et al., 2021; Yan et al., 2022a; Sun et al., 2023a; Sun et al., 2023b; Liao et al., 2023; Peng et al., 2023; Wu et al., 2023)	Male C57BL/6 mice, NIT-1 cells (Sun et al., 2023a)		Pancreatic beta cell: GPX4↑, ACSL4↓
	C57BL/6 (wild-type, WT) mice, Neonatal rat cardiomyocytes (Liao et al., 2023); Sprague-Dawley rats, H9c2 cells (Wu et al., 2023)		Cardiomyocyte: AMPKα2↑(80, 81)
	Human colonic tissue samples, Male C57BL/6 mice (Sun et al., 2023b)		Intestinal epithelial cells: AMPK↑
	Male Sprague-Dawley (SD) rats, VSMCs (Ma et al., 2021)		Vascular endothelial cell: Nrf2↑
	C57 BL/6J female mice (Peng et al., 2023)		Ovaries of mice: GPX4/mTOR/SIRT3↑
	adult male C57BL/6 mice (Yan et al., 2022a)		Chondrocyte: GPX4↑, ACSL4↓
Dapagliflozin (Huang et al., 2022)	C57BL/6 mice		Renal tubular cell: reduce ubiquitination degradation of FPN1
Canagliflozin (Ma et al., 2022)	Male DSS rats		Cardiomyocyte: TfR1, ACSL4, NOX4↓; GSH, FTH1↑
Nutraceutical approaches			
Silybin (Song et al., 2022b; Yan et al., 2022b)	HepG2 and HL7702 cells (Song et al., 2022b)	Reverses ferroptosis	
	HepG2 cells (Yan et al., 2022b)	Combine with TfR1 to reduce iron intake, ACSL4↓	
Vitamin E (Carlson et al., 2016; Zhang et al., 2022c)	Male Sprague–Dawley (SD) rats (Zhang et al., 2022c)		Neurons: 15-LOX↓; GSH, GPX4↑
	Hepatocyte-specific mice (Carlson et al., 2016)	15-LOX↓ (collaborate with GPX4)	
Vitamin D (Cheng et al., 2021b; Zhao et al., 2022a)	Male Zucker lean (ZL) rats, Islet *β* (INS-1) cells (Zhao et al., 2022a)		Pancreatic beta cell: nuclear factor kappa-B, DMT1↓
	Zebrafish liver cells (Cheng et al., 2021b)	Keap1-Nrf2-GPX4↑; nuclear factor kappa-B-hepcidin↓	
Fish oil (Shi et al., 2022a; Wang et al., 2022d)	Male Wistar rats (Shi et al., 2022a)		Vascular endothelial cell: SLC7A11↑
	PTZ kindling mice (Wang et al., 2022d)		Neurons: Nrf2↑
Nutraceutical approaches			
Astaxanthin (Luo et al., 2022b; Cai et al., 2022)	RAW264.7 cells (Luo et al., 2022b)		RAW264.7cell: Nrf2/HO-1↑
	Male C57BL/6 mice, human hepatic, L-02 cells (Cai et al., 2022)	Nrf2/HO-1↑	
Resveratrol (Zhang et al., 2022d; Wang et al., 2022e)	MIN6 cells (Zhang et al., 2022d)		Pancreatic beta cell: endoplasmic reticulum stress↓; PPAR-γ↑
	Male Kunming mice (Wang et al., 2022e)	DMT1, TfR1↓; FPN1↑	
Berberine (Bao et al., 2023; Song et al., 2023)	Islet *β* cells (Bao et al., 2023)		Pancreatic beta cell: GPX4↑
	H9c2 cells (Song et al., 2023)		Myocardial cell: TfR1, P53↓; Nrf2/HO-1, FTH1, GPX4↑
Green tea extract (Kose et al., 2019; Ding et al., 2023)	MIN6 cells (Kose et al., 2019)		Pancreatic beta cell: GSH, GPX4↑
	Male C57BL/6 mice (Ding et al., 2023)	GSH, GPX4↑; ACSL4↓	
Curcumin (Kose et al., 2019; Tang et al., 2021; Wei et al., 2022; Sun et al., 2023c)	MIN6 cells (Kose et al., 2019)		Pancreatic beta cell: GSH, GPX4↑
	Male New Zealand rabbits, Rat H9C2 cardiomyocytes (Wei et al., 2022)	Nrf2/HO-1, GPX4↑	
	Human bronchial epithelial cell line BEAS-2B, male Sprague-Dawley rats (Tang et al., 2021)		Pulmonary epithelial cell: SLC7A11, FTH1, GPX4↑; TfR1↓
	TX mice, Rat normal liver cells (BRL-3A) (Sun et al., 2023c)		Myocardial cell: Nrf2↑
Other drugs that are effective in clinical trials			
Bicyclol (Zhao et al., 2022b)	Male C57BL/6 mice, Normal human hepatocytes L-O2	Nrf2, GPX4↑	
Fibroblast growth factor 21 (Wu et al., 2021)	C57BL/6J male mice	Nrf2/HO-1↑	
Obeticholic acid (Cao et al., 2023)	Female C57BL/6 mice		Uterus of mice: GPX4, SLC7A11↑

Note: ↑ represents target activation, increased expression, and upregulation of pathways; ↓ indicates target inhibition, reduced expression, and downregulation of pathways.

Currently, hypoglycemic drugs, such as glucagon-like peptide-1 receptor agonists (GLP-1RA) and thiazolidinedione (TZDs), have been recommended as treatment options for MASLD in guidelines (Chalasani et al., 2018; Tokushige et al., 2021). Although metformin, sodium-glucose linked transporter 2 (SGLT2) inhibitors, along with DPP4 inhibitors have shown certain efficacy in clinical trials, their strength of evidence has remained insufficient for first-line drugs. TZDs have been identified as inhibitors of ferroptosis (Doll et al., 2017; Kung et al., 2022), predominantly by inhibiting the activity of ACSL4, thereby attenuating the enzymatic conversion of unsaturated fatty acids. Studies have shown that rosiglitazone can improve arsenic-induced ferroptosis in hepatocytes by targeting ACSL4 (72). In neurons, pioglitazone exhibits anti-ferroptosis activity in neurons by increasing the expression of PPAR-γ, downregulating cyclooxygenase-2 (COX2) expression (Liang et al., 2022), and cooperating with Nrf2(74). Furthermore, pioglitazone and mitoglitazone were found to target CISD1 through the stabilizing Fe-S clusters, thereby alleviating mitochondrial ferroptosis (Yuan et al., 2016b; Qi et al., 2023). Liraglutide has also demonstrated a delay in fatty liver progression in db/db mice through various aspects, including the improvement of iron metabolism and GPX4 activity (Song J-X. et al., 2022). Similar anti-ferroptosis mechanisms of liraglutide have been observed in neurons (An et al., 2022) as well. Although the research on the impact of metformin, DPP4 inhibitors, and SGLT2 inhibitors on liver ferroptosis is limited, existing fundamental studies indicate their potential influences on various cellular systems. For instance, metformin has been demonstrated to inhibit ferroptosis in various conditions, including pancreatic β cell injury, cardiac ischemia/reperfusion, colitis, osteoarthritis, polycystic ovary syndrome, as well as hyperlipidemia-associated vascular calcification. The underlying mechanisms involve the activation of AMPK and Nrf2 pathways (Ma et al., 2021; Yan J. et al., 2022; Sun Y. et al., 2023; Sun SP. et al., 2023; Liao et al., 2023; Peng et al., 2023; Wu et al., 2023). Vildagliptin has demonstrated the ability to upregulate the expression of GPX4 and improve ferroptosis in neurons (Zhang Y. et al., 2022), suggesting its possible inhibitory impact on DPP4 similar to p53 (87). SGLT2 inhibitors have been found to play an anti-ferroptosis role in renal tubular cells of diabetic nephropathy mice by combining with FPN1 to reduce its ubiquitination degradation (Huang et al., 2022), as well as in cardiomyocytes of heart failure mice (Ma et al., 2022). Further research is imperative to fully understand the potential roles and mechanisms of these pharmaceutical agents pertaining to the selective targeting of ferroptosis within the context of MASLD.

As reported by the International Lipid Expert Group in 2023, the beneficial clinical evidence of various nutritional supplements in the context of nutritional food therapy for MASLD have been highlighted (Rizzo et al., 2023). These supplements include vitamin D, vitamin E, silymarin, green tea extract, curcumin, fish oil, berberine, and resveratrol, et al. These supplements have shown efficacy in delaying the progression of MASLD and regulating ferroptosis in basic research. Some of these supplements may also provide benefits for individuals with both diabetes and fatty liver (Table 1).

Moreover, several endogenous target active molecules and biological analogues have progressed to clinical phase II-III trials. Examples include fibroblast growth factor 21 analogues (FGF21) (Luo Y. et al., 2022) and the FXR agonist obeticholic acid (Younossi et al., 2019b). Research has demonstrated the involvement of FGF21 and FXR agonist in the regulation of ferroptosis. These emerging therapeutic options hold promise for the management of MASLD.

## 5 Other active substances targeting ferroptosis in basic research

In the realm of basic research, various active components and endogenous substances found in drugs have demonstrated potential in improving ferroptosis associated with metabolic diseases and fatty liver. These substances hold promise as key drug molecules, acting on relevant targets to ameliorate symptoms of MASLD. Additionally, the continuous discovery of novel targets further expands our understanding in this field. We summarized the effective active ingredients in Table 2.

**TABLE 2:** The mechanism of ferroptosis targeted by other active substances in the basic research stage.

Active substance/Drug	Cell/Model	Target and effect
Active substances that act on both pancreatic beta cells and liver cells		
Iron chelator (Deferiprone, Deferoxamine) (Chen et al., 2022a)		Chelate iron and inhibit lipid peroxidation
Ferrostatin-1 (Jiang et al., 2022a)		Regulating iron metabolism
Quercetin (Li et al., 2020c; Jiang et al., 2022b)	Pancreatic beta cells of C57BL/6J mice with diabetes	MDA↓; GSH, VDAC2↑
	COX2, ACSL4, mitochondrial ROS↓; GPX4↑	steatotic L-02 cells, C57BL/6J mice
Other active substances		
Ginkgolide B (Yang et al., 2020)	ApoE−/− mice, HepG2 cells	Nrf2↑
Dehydroabietic acid (Gao et al., 2021)	Fatty liver mice	Nrf2↑
Leonine (Salama et al., 2022)	Wistar rats	Nuclear factor kappa-B↓; Nrf2↑
Thymosin beta 4 (Zhu et al., 2021)	L-02 cells	GPX4↑
dimethyl fumarate (Zhang et al., 2020)	C57BL/6 mice, HepG2 cells/L-02 cells	Nrf2↑
fucoidan (Xue et al., 2022)	Sprague-Dawley rats	DMT1, FPN1↓; p62, Nrf2, SLC7A11, GPX4↑
glycyrrhizin (Wang et al., 2019)	L-02 cells	Nrf2/HO-1, GPX4↑
Betaine (Li et al., 2022)	C57BL/6 mice	Stable ZIP14, FPN1
d-Cysteine (Homma et al., 2022)	Hepa 1–6 cells	GSH↑
Apigenin (Han et al., 2022)	AML12 cells	GPX4↑
Ulinastatin (Wang et al., 2021b)	L-02 cells, C57BL/6 mice	Sirt1/Nrf2/HO-1↑
VBIT-12 (Niu et al., 2022)	C57BL/6J mice	Inhibition of mitochondrial VDAC1 oligomerization
Taurine (Zhang et al., 2014)	Male Kunming mice	GSH, GPX4↑
Schisandrin B (Shi et al., 2022b)	SD rats	Nrf2, GPX4↑; NOX2/4↓
Gingerenone A (Chen et al., 2022b)	HepG2 cells	Nrf2,-GPX4↑
Oleanolic acid (Ouyang et al., 2023)	C57BL/6 male mice	GPX4, SLC7A11↑; TfR1↓
Atractylodin (Ye et al., 2023)	C57BL/6J mice	Nrf2, GPX4, SLC7A11, FTH1↑

Note: ↑ represents target activation, increased expression, and upregulation of pathways; ↓ indicates target inhibition, reduced expression, and downregulation of pathways.

## 6 Discussion and summary

In summary, it is evident that metabolic diseases and fatty liver share a close relationship and mutually influence each other as risk factors. Both conditions are characterized by iron overload and ferroptosis. Consequently, ferroptosis indirectly contributes to the interplay between metabolic diseases and fatty liver, promoting the onset and progression of MASLD.

Regarding drug treatment, numerous clinical studies emphasize the significance of hypoglycemic drugs as essential therapeutic agents for managing MASLD. These drugs exhibit potential anti-ferroptotic effects in various cell types and align with the previously discussed key targets. As a result, they offer promise in combating ferroptosis. Moreover, apart from their intrinsic hypoglycemic effects, these drugs serve as an ideal option for comprehensive control of MASLD occurrence and progression. Furthermore, ongoing clinical research investigating nutritional supplements and active ingredients derived from traditional Chinese medicine has demonstrated effective anti-ferroptotic properties. These therapeutic approaches present notable advantages and should not be underestimated. Considering the multifaceted nature of MASLD the identification of effective drugs, such as hypoglycemic agents, for both metabolic diseases and MASLD could yield promising therapeutic outcomes in controlling risk factors and providing targeted treatment. Additionally, we recommend an increased assessment of serum iron metabolism markers in future clinical trials involving individuals with MASLD, particularly when evaluating the effects of the aforementioned drugs. This approach will enable a more thorough investigation into the potential effectiveness of these drugs in mitigating ferroptosis.

This article summarizes the possible relationship between MASLD and related metabolic diseases and ferroptosis. Research has shown that ferroptosis affects multiple signaling molecules and pathways in the body, leading to metabolic disorders and inducing MASLD. Ferroptosis inhibitors can alleviate liver steatosis. It can be inferred that ferroptosis may be an important pathogenesis for the occurrence and development of MASLD. The article summarizes the relevant targets or pathways of MASLD therapeutic drugs acting on ferroptosis, as well as the mechanisms and pathways of targeted drugs/active substances inhibiting ferroptosis, in order to explore the process and form of ferroptosis in MASLD. At present, there is still a lack of further research to elaborate on the specific mechanisms by which these drugs or active substances affect the progression of MASLD through ferroptosis. The pathways of ferroptosis-induced diseases are complex and diverse, with different therapeutic drugs targeting different targets. Perhaps multi-drug combinations or ferroptosis multi-pathway inhibitors can more effectively reverse MASLD. In addition, more clinical studies on MASLD drugs are needed to confirm that inhibition of ferroptosis can improve MASLD in clinical practice. We believe that more research will reveal the regulatory mechanisms of ferroptosis in the future, providing strong evidence for targeted ferroptosis prevention and treatment of MASLD.

## Figures and Tables

**Table:** 

**ALT**	alanine transaminase
**AMPK**	AMP activated protein kinase
**BH4**	tetrahydrobiopterin
**CISD1**	CDGSH iron sulfur domain 1
**CoQ**	coenzyme Q
**DHFR**	dihydrofolate reductase
**DHODH**	dihydroorotate dehydrogenase
**DIOS**	dysmetabolic iron overload syndrome
**DMT1**	divalent metal transporter 1
**DPP4**	dipeptidyl peptidase 4
**Fer-1**	ferrostatin-1
**FGF21**	fibroblast growth factor 21 analogues
**FPN1**	ferroportin 1
**FSP1**	ferroptosis suppressor protein 1
**FTH1**	ferritin heavy chain 1
**FtMt**	mitochondrial ferritin
**FXR**	farnesoid X receptor
**GCH1**	guanosine triphosphate cyclization Hydrolase 1
**GLP-1RA**	glucagon-like peptide-1 receptor agonist
**GPX4**	glutathione peroxidase 4
**GSH**	glutathione
**HO-1**	heme oxygenase 1
**IL**	interleukin
**keap1**	kelch-like epichlorohydrin-associated protein-1
**LIP**	liable iron pool
**LOX**	lipoxygenase
**LPCAT3**	lysophosphatidylcholine acyltransferase 3
**MAFLD**	metabolic dysfunction-associated fatty liver disease
**MASH**	metabolic dysfunction-associated steatohepatitis
**MASLD**	metabolic dysfunction-associated steatotic liver disease
**MDA**	malondialdehyde
**mTOR**	mechanistic target of rapamycin
**MtROS**	mitochondrial reactive oxygen species
**NASH**	non-alcoholic steatohepatitis
**NCOA4**	nuclear receptor coactivator 4
**Nrf2**	nuclear factor erythroid 2-related factor 2
**OH-**	hydroxide ion
**PCBP1**	poly (rC) binding protein 1
**PL**	phospholipid
**PPAR**	peroxisome proliferator activated receptor
**PUFA**	polyunsaturated fatty acid
**ROS**	reactive oxygen species
**SGLT2**	sodium-glucose linked transporter 2
**SLC7A11**	solute carrier family 7 member 11
**STEAP3**	six-transmembrane epithelial antigen of the prostate 3
**STZ**	streptozotocin
**Tf**	transferrin
**TfR1**	transferrin receptor 1
**TNF-α**	tumor necrosis factor *a*
**TZD**	thiazolidinedione
**T2DM**	type 2 diabetes mellitus
**VDAC**	voltage dependent anion channel
**ZIP8/14**	zinc transporter 8/14
**4-HNE**	4-hydroxynonenal
